# Genome‐scale metabolic modelling of extremophiles and its applications in astrobiological environments

**DOI:** 10.1111/1758-2229.13231

**Published:** 2024-01-09

**Authors:** Nuttapol Noirungsee, Sakunthip Changkhong, Kittiya Phinyo, Chutipong Suwannajak, Nahathai Tanakul, Sahutchai Inwongwan

**Affiliations:** ^1^ Department of Biology, Faculty of Science Chiang Mai University Chiang Mai Thailand; ^2^ Research Center of Microbial Diversity and Sustainable Utilizations, Faculty of Science Chiang Mai University Chiang Mai Thailand; ^3^ Department of Thoracic Surgery University Hospital Zurich Zurich Switzerland; ^4^ Research group on Earth—Space Ecology (ESE), Faculty of Science Chiang Mai University Chiang Mai Thailand; ^5^ Office of Research Administration Chiang Mai University Chiang Mai Thailand; ^6^ National Astronomical Research Institute of Thailand Chiang Mai Thailand

## Abstract

Metabolic modelling approaches have become the powerful tools in modern biology. These mathematical models are widely used to predict metabolic phenotypes of the organisms or communities of interest, and to identify metabolic targets in metabolic engineering. Apart from a broad range of industrial applications, the possibility of using metabolic modelling in the contexts of astrobiology are poorly explored. In this mini‐review, we consolidated the concepts and related applications of applying metabolic modelling in studying organisms in space‐related environments, specifically the extremophilic microbes. We recapitulated the current state of the art in metabolic modelling approaches and their advantages in the astrobiological context. Our review encompassed the applications of metabolic modelling in the theoretical investigation of the origin of life within prebiotic environments, as well as the compilation of existing uses of genome‐scale metabolic models of extremophiles. Furthermore, we emphasize the current challenges associated with applying this technique in extreme environments, and conclude this review by discussing the potential implementation of metabolic models to explore theoretically optimal metabolic networks under various space conditions. Through this mini‐review, our aim is to highlight the potential of metabolic modelling in advancing the study of astrobiology.

## Graphical abstract

Metabolic modelling with GEMs is a valuable tool for studying extremophiles and simulating genetic and environmental changes. Integration with other mathematical approaches aids in predicting life forms in astroenvironments, informing the origins of life and space adaptation. Challenges remain, necessitating research and innovative data collection for the applications in astrobiology.

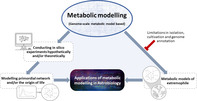

## INTRODUCTION

Astrobiology is the study of life in the universe based on biological concepts related or applicable to astronomical contexts. Astrobiology extends beyond the study of extra‐terrestrial life and encompasses an astrobiological perspective on life existing on Earth (Hallsworth et al., 2021). Unlike its overlapping field, exobiology, astrobiology does not only focus on searching for extra‐terrestrial life, but also includes the studies of the origin of life and extremophilic organisms (Chyba & Hand, 2005). Understanding how life has emerged, distributed and adapted to various extreme conditions could help prospect the potentially habitable environments in the universe, and determine the possible characteristics favouring life in the space conditions (Des Marais et al., 2008). Most extra‐terrestrial environments would be considered extreme in an anthropocentric perspective due to the degrees of physicochemical parameters divergent from the conditions that most organisms can survive. The studies of astrobiology could shed light on the diverse habitats and interactions of extremophile microbes, contributing to our understanding of Earth's biosphere and the adaptations of organisms to overcome environmental constraints, which is one of the most conspicuous steps for astrobiological studies.

The group of organisms known as extremophiles can survive and thrive on the extreme ends of the environmental conditions considered too harsh. For example, thermophiles survive at temperatures as high as 122°C (higher than the temperature mostly used for sterilization). Psychrophiles subsist at subzero temperatures (Amato et al., 2010). Piezophiles flourish under the bone‐crushing pressures of the Mariana Trench (Rampelotto, 2013). Understanding how extremophiles cope with conditions analogous to potential habitable extra‐terrestrial environments, such as spaceflight or conditions on celestial bodies, is essential for prospecting for extra‐terrestrial life and speculating on its potential genesis in such environments (Mastascusa et al., 2014; Seckbach & Oren, 2001; Smith et al., 2009; Thombre et al., 2020). Research analysing omics data obtained from spaceflight and corresponding terrestrial analog experiments has garnered scientific interest. A notable repository for such data is NASA's GeneLab, which archives information on extremophilic microbes, including heat‐shock tolerant Firmicutes belonging to Bacillales and Micrococcales such as *Alkalihalobacillus gibsonii*, and *Alkalihalobacillus clausii*. The database also contains a range of model organisms, including animals like *Caenorhabditis elegans* (roundworm) and *Mus musculus* (mouse), as well as plants such as *Arabidopsis thaliana* (thale cress). These studies are directed towards elucidating the biological responses to conditions encountered in space, from microgravity to elevated levels of radiation (NASA GeneLab: Open Science for Life in Space, 2022). However, the application of systems biology approaches and genome‐scale metabolic network modelling, a technique for predicting metabolic phenotypes, is highly limited in this context.

In this concise review, we provide an overview of the applications and advantages of metabolic modelling in the study of extremophilic microbes in space‐related environments. We summarize the current state of the art in metabolic modelling approaches within the astrobiological context, including its theoretical exploration of the origin of life in prebiotic environments and the utilization of genome‐scale metabolic models for extremophiles. We also highlight the challenges associated with applying this technique in extreme environments. With the potential implementation of metabolic models to investigate theoretically optimal metabolic networks under space conditions, we hope to emphasize the significant potential of metabolic modelling in advancing astrobiology research.

## METABOLIC MODELLING AND ITS ADVANTAGES IN ASTROBIOLOGY

Metabolic modelling is a powerful tool in systems biology that allows for the study of a metabolic network as a whole. At the centre of an organism, the metabolic network plays crucial roles in cellular processes, transforming nutrients into energy and building blocks for cellular biomass. Understanding the systemic features of the metabolic network provides insight into metabolic phenotype, capacity, flexibility, and stability of the organisms, which determine the responsive mechanism and adaptive ability to the environment (Kuepfer, 2014). The genome‐scale metabolic model (GEM), a mathematical model based on the stoichiometry of all metabolic reactions in a cell, has been used to quantify the metabolic phenotypes of various organisms. This approach has become a powerful tool in systems biology and synthetic biology to optimize the production of targeted metabolites. The steps of reconstructing GEM involve drafting a reconstruction based on genomic data of an organism of interest; many tools have been designed to facilitate this process such as the RAVEN toolbox, KBASE, PathwayTools and the ModelSEED (Thor et al., 2017). The following iterative processes of manual curation and refinement of the reconstruction can continue until the model is evaluated and validated to be informative depending on the objective of the reconstruction (Thiele & Palsson, 2010).

To utilize the stoichiometric model, the standard approach is flux balance analysis (FBA), which is based on the tabulation of metabolic flux in numerical matrix form constrained by the stoichiometry and biomass composition of the cells (Orth et al., 2010). The linear programming algorithm provides the analysis of optimal solutions for FBA, such as the Constraint‐Based Reconstruction and Analysis (COBRA) toolbox, which is widely used in this analysis (Kuepfer, 2014). Some common limitations of FBA include the prerequisite of metabolic steady state and the complexity to identify the optimal objective function (García Sánchez & Torres Sáez, 2014). Several extensive approaches were developed from the addition and/or modification of constraints based on empirical or mechanical evidences to improve the accuracy and precision of the metabolic prediction by FBA. These approaches include Genome‐scale models with Enzymatic Constraints using Kinetic and Omics data (GECKO). GECKO takes enzyme concentrations as variables to account for enzymes' limited catalytic activity (Sánchez et al., 2017). Another approach is MetabOlic Modelling with eNzyme kineTics (MOMENT), which includes enzyme turnover rates and enzyme molecular weights (Adadi et al., 2012). Additionally, thermodynamic‐based flux balance analysis (TFA) is utilized. TFA couples reaction directionality with Gibbs free energy to eliminate thermodynamically infeasible predictions (Henry et al., 2007; Soh & Hatzimanikatis, 2014). In addition, numerous studies have tried to integrate omics data with GEM. One such approach is the Metabolic and Expression Models (ME‐models), which includes metabolic, catalytic, and cellular expression restrictions. FlexFlux is based on seeking steady‐states of regulatory networks and combines the analysis of both regulatory networks and FBA (Lerman et al., 2012; Marmiesse et al., 2015; O'Brien et al., 2013; Salvy & Hatzimanikatis, 2020).

With the extensive functions mentioned above, metabolic modelling demonstrates the great capacity and potential advantages for study organisms in silico. GEMs can precisely identify metabolic targets or significant pathways that respond to different environmental conditions. Unlike genomic and transcriptomic analysis, GEMs have the capability to predict cellular behaviour by simulating metabolic fluxes (O'Brien et al., 2015). This enables researchers to understand how cells respond to varying conditions such as nutrient availability or stress. For example, a genomic analysis and GEM study was conducted on *Alicyclobacillus tolerans*, an iron‐oxidizing bacterium isolated from an acid mine drainage. The curated GEM accurately predicted the bacterium's growth characteristics and its ability to utilize diverse carbon sources. Additionally, the analysis revealed a significant presence of genes associated with metal resistance, indicating the bacterium's robust adaptation to extreme environments (Enuh & Aytar Çelik, 2022). Another study on GEMs of *Microbacterium* species, CGR1 and CGR2, isolated from the Atacama Desert, reported conserved metabolic pathways and attributes, highlighting their adaptation to the environment. Notably, CGR1 exhibited greater connectivity of specific metabolites related to pH tolerance and CO_2_ production, suggesting its role in handling acidic stress within a wider pH tolerance range. Both strains were predicted to synthesize pigment metabolites, which was confirmed through HPLC experiments (Mandakovic et al., 2020). These studies provide valuable knowledge in the metabolic adaptations of extremophiles to various abiotic factors within extreme environments. They serve as a valuable resource for further investigations, including the study of astrobiology.

Furthermore, in silico experimentation using GEMs provides the capability to conduct hypothetical or theoretical experiments. GEMs allow for testing hypotheses about cellular metabolism by simulating the effects of genetic perturbations or environmental changes. They also enable in silico experimentation, where metabolic engineering strategies can be simulated, and outcomes can be predicted before conducting actual experiments, particularly in challenging experimental conditions. Additionally, these in silico experiments can be modified and analysed with the inclusion of additional methods and constraints to be specific to the objective or currently available evidence/knowledge. For example, factors like air composition and radiation effects could be included in the analysis, and incorporating enzymatic kinetics under different conditions could help improve accuracy, as mentioned (Adadi et al., 2012; Sánchez et al., 2017). Moreover, this modelling approach possesses the capability to incorporate various parameters characterizing the physical, chemical, and genomic attributes of distinct astrobiological settings, such as Enceladus, one of Saturn's moons (Affholder et al., 2022). This integration offers the potential to forecast the possible presence of life forms within this environment. These extensive methods help enhancing predictive power and expanding the scope of studies to environments that are difficult to explore in vivo or in vitro.

## METABOLIC MODELLING IN ASTROBIOLOGY CONTEXT

Metabolic modelling has become an essential tool in modern biology for predicting the metabolic phenotypes of organisms and identifying potential metabolic targets for engineering (García‐Jiménez et al., 2021). Although metabolic modelling has found extensive applications in industry, its potential in astrobiology remains largely unexplored. Recently, a study delved into the interactions among microbes within the International Space Station (ISS) microbiome, employing advanced metabolic modelling techniques. This investigation focused on the prevalent presence of *Klebsiella pneumoniae*. Through computational models, the research discerned crucial metabolic interactions and dependencies, shedding light on the beneficial interactions of *K. pneumoniae* with other microbes, while also uncovering parasitic interactions with *Aspergillus* and *Penicillium* species. These findings underscore the significance of *K. pneumoniae* within the ISS microbiome and the potential for understanding similar microbiomes in alternative environments (Kumar et al., 2022). One area of interest is the theoretical prediction of life in space conditions using metabolic modelling. By exploring how metabolic modelling can be used to study theoretical biology and the origins of life on Earth and possibly elsewhere, researchers hope to uncover the fundamental biological processes that could lead to the emergence and survival of life in extra‐terrestrial environments (Xavier et al., 2021). This is particularly important given the growing interest in space exploration and the search for life beyond Earth (Merino et al., 2019; Schultz et al., 2023). Metabolic modelling can also be used to study the metabolic processes of organisms in space and in prolonged extreme conditions (Kumar et al., 2022). For example, by studying the genomes and metabolic networks of prokaryotic extremophiles, researchers can gain investigate into how these organisms have adapted to survive in extreme environments (Table 1 and references therein). This information can then be used to inform the design of metabolic pathways that could be used to sustain life in space (Weber et al., 2023). Another potential application of metabolic modelling in astrobiology is the production of compounds for use in space (Sharma & Curtis, 2022). For example, metabolic engineering could be used to produce valuable compounds such as drugs or materials in space environments where traditional production methods are not feasible (Benoit et al., 2006; De Gelder et al., 2009).

**TABLE 1:** Summary of published genome‐scale metabolic models of extremophiles.

Extremophilic group	Metabolism	Species	No. reaction	No. metabolite	Ref.
Acidophiles	Chemoorganotrophic	*Alicyclobacillus tolerans*	2187	1468	(Enuh & Aytar Çelik, 2022)
		*Microbacterium* sp. CGR1	1168	904	(Mandakovic et al., 2020)
Alkaliphiles	Methanotrophic	*Methylotuvimicrobium alcaliphilum*	432	422	(Nguyen et al., 2020)
	Chemoorganotrophic	*Microbacterium* sp. CGR1,2	1172	897	(Mandakovic et al., 2020)
Halophiles	Chemoorganotrophic	*Chromohalobacter salexigens*	1530	1123	(Piubeli et al., 2018)
		*Chromohalobacter salexigens* DSM 3043	1387	1411	(Ates et al., 2011)
		*Halobacterium salinarum*	644	545	(Gonzalez et al., 2009)
		*Halomonas smyrnensis* AAD6T	1142	980	(Diken et al., 2015)
		*Salinibacter ruber*	1459	1363	(Bagheri et al., 2019)
Psychrophiles	Chemoorganotrophic	*Pseudoalteromonas haloplanktis* TAC125	1322	1133	(Fondi et al., 2015)
Thermophiles	Methanogenenic	*Methanococcus jannaschii*	609	510	(Tsoka et al., 2004)
	Acetogenic	*Moorella thermoacetica*	705	698	(Islam et al., 2015)
	Chemoorganotrophic	*Brevibacillus thermoruber* 423	1454	1410	(Yaşar et al., 2019)
		*Geobacillus icigianus*	1676	1589	(Kulyashov et al., 2020)
		*Parageobacillus thermoglucosidasius* C56‐YS93	1159	1163	(Ahmad et al., 2017)
		*Parageobacillus thermoglucosidasius* NCIMB1195	1175	890	(Mol et al., 2021)
		*Thermatoga* sp. Strain RQ7	692	538	(Gautam & Xu, 2021)
		*Thermus thermophilus*	75^a^	73^a^	(Swarup, 2010)
		*Thermus thermophilus* HB27	796	635	(Lee et al., 2014)
Polyextremophiles	Chemoorganotrophic	*Shewanella piezotolerans*	922	653	(Dufault‐Thompson et al., 2017)
	Chemoorganotrophic/chemoautotrophic	*Sulfolobus solfataricus*	776	705	(Ulas et al., 2012)
Satellotrophs^a^	Various	International Space Station (ISS) Microbiome	Consisting of 52 bacterial GEMs		(Kumar et al., 2022)

^a^ Microorganisms that inhabit artificial satellites.

In astrobiology‐related context, metabolic modelling can be used to theoretical explore life in various geochemical components. FBA with chemical energy integrated was used to predict the microbial kinetic in conditions of interest (Shapiro et al., 2018), and a stochiometric model theoretically predicted the emergence of protometabolism in the absence of phosphate (Goldford et al., 2017). Alternatively, kinetic modelling integrates kinetic parameters of the enzymes in the metabolic network into the analysis, providing the model more predictive power compared to FBA of GEMs, especially for the well‐defined sections in the network. Unlike FBA, based on the kinetic parameters, kinetic models can predict the level of metabolites and fluxes under either metabolic steady or non‐steady state (Hameri et al., 2019; Islam et al., 2021). With adequate information, kinetic modelling could become one of the most powerful tools to theoretically study metabolic network in space‐related aspect. For instance, the model could be combined with the kinetic studies of the formation of organic matters in extra‐terrestrial conditions (Kebukawa & Cody, 2015; Tan & Sephton, 2021; Zhang et al., 2010) to predict the initial states of origin of life. An isotopologue‐specific kinetic model was constructed to comparatively predict the quantities of isotopologue signatures of methane generated by biological‐ and metal‐catalysed CO_2_ methanation (Cao et al., 2019). With the integration of geochemical properties, thermodynamic and kinetic modelling were used to predict the habitability the ocean–seafloor system on Enceladus (Hao et al., 2022). However, the main challenge for kinetic modelling is the study of biochemical networks at organismic level, the major limitation of this approach is the difficulty in the analysis of wholistic regulatory process and the lack of the available kinetic parameter network (Kruger & Ratcliffe, 2015). With model‐driven hypotheses, metabolic modelling could play a key role in the theoretical study of the metabolic network, the application of metabolic modelling in astrobiology could utilize the ideas of theoretical modelling of the origin(s) of life and the analysis of adaptive pathways of organisms in space‐related conditions.

### Metabolic modelling of prebiotic and primordial life

Astrobiology also investigates the processes of prebiotic synthesis of organic molecules, and the evolution of the building blocks of life, or essentially, the primordial stage of life itself (Petrignani & Candian, 2022). To become a living thing, the chemical reactions need to connect and function as a network, known as a metabolic network, which regulates and determines the physiological processes of an organism. Since the idea that all life on Earth has a common origin become well‐established only in the twentieth century, different perspectives of how life began have been proposed. The early Earth had a similar history and conditions to those of Mars, Venus, Europa and Enceladus etc., understanding the processes of the origin(s) of life on Earth may help prospect the existence of life beyond the confines of Earth or even in the extra‐terrestrial environments (Kumar et al., 2020). Before the emergence of life, the synthesis of various compounds is considered an essential initial step. The synthesis of these early complex molecules is not expected to be assisted by enzymes as catalysts or resemble the characteristics of modern metabolic networks. This idea led to investigating the nonenzymatic synthesis of biochemical molecules under early Earth conditions, hypothesizing that life began with a pool of the primordial nonenzymatic metabolic reactions which have the function to build and break down the building block of the cell (Muchowska et al., 2020).

Evidently, ‘life as we know it’ is a lot more complex than a set of prebiotic chemical reactions, but the study of how biomolecules are formed in the specific condition, like extra‐terrestrial conditions, could pave our way to the understanding on how life may be like in that environment (Harrison & Lane, 2018). The study of the evolution of central metabolism suggests that the network could have been constructed under the environment of the prebiotic Archean Ocean. The potential chemical conditions were generated based on the composition of sediments from the geologic era to examine the formation of metabolites found in the modern metabolic networks. The study reported that the sequences of reactions involved in central carbon metabolism, glycolysis and pentose phosphate pathways, could have been catalysed by the iron‐rich oceanic environment (Keller et al., 2014).

The metabolic modelling approaches have been seldomly used to investigate the primordial chemical networks and the origin of life. To understand the universally essential metabolic needs of prokaryotes, an integrative study of biomass constituents in 71 manually curated GEMs and a diverse set of publications in gene essentiality and enzyme‐cofactor association provides insight into the minimal required cofactors for the prokaryotic metabolic networks (Xavier et al., 2017). A study of essential metabolic reactions for prokaryotes analyses 15 GEMs, integrated with 36 large‐scale gene essentiality datasets from a broad range of bacterial and archaeal species, and the conserved metabolic genes estimated from 79 prokaryotic genomes. The results demonstrate that essential genes are highly conserved for several species, and tRNA‐charging module and cofactor metabolism are found to be ancestral and central to the metabolic networks (Xavier et al., 2018). Furthermore, to speculate the metabolic features of the last bacterial common ancestor (LBCA), 1089 anaerobic bacterial genomes were collated and translated into 146 protein families which are expected to be conserved in the core metabolic network of LBCA. The results indicate that only nine genes are required to confine universal metabolites, and LBCA utilized the multifunctional enzymes from RNA modifications (Xavier et al., 2021).

### Genome‐scale metabolic modelling of extremophiles and its challenges

In the exploration of life in extra‐terrestrial contexts, a common and preliminary strategy involves investigating the characteristics of nearby environments and extrapolating this knowledge to other planetary bodies. The examination of life thriving under extreme conditions and pushing the boundaries of habitability on Earth can lead to the perception of the potential existence and locations of extra‐terrestrial life forms. For example, Taubner et al., 2018 investigated the growth of different thermophilic methanogens under Enceladus‐like conditions in order to illustrate the possible modes of life on the Saturn's icy moon, which is one of the prime targets in the search for life in the solar system (Hao et al., 2022). Similarly, Mastascusa et al., 2014 used a Martian environment Simulator to investigate the ability of four extremophilic microorganisms to survive the temperatures, radiation, desiccation and pressures analogous to Mars. While simulations on Earth attempt to mimic the conditions of Mars or other celestial bodies, they fall short in capturing every aspect of these extra‐terrestrial environments. For instance, the levels of cosmic radiation present on the actual celestial bodies are challenging to replicate accurately on Earth (Huff et al., 2023). The study of thermophiles diversity in New Zealand's hot spring stromatolitic digitate sinters, whose morphology is similar to opaline silica rocks found by the Spirit rover, provides the perspectives of potential life that might once live on Mars (Sriaporn et al., 2020).

The extra‐terrestrial contexts are, nevertheless, not limited to earthly extreme conditions hostile to humans. Spaceflight conditions present a unique set of challenges including microgravity and space radiation (da Silveira et al., 2020; Durante & Cucinotta, 2008; Garrett‐Bakelman et al., 2019). As a result, organisms that are not considered as extremophiles have demonstrated the ability to adapt and thrive in such environments. For example, proteomic analysis of *Escherichia coli* aboard a spacecraft for a duration of about 400 hours showed changes associated with amino acid and fatty acid metabolism, albeit no changes in haemolysis, morphology, or antibiotic sensitivity were detected (Zhang et al., 2015). Transcriptomic and proteomic studies revealed responses of *Pseudomonas aeruginosa* to spaceflight conditions through regulation of genes relating to growth under anaerobic conditions (Crabbé et al., 2011). The applications of omics approach on elucidating molecular mechanisms of microbial survivability in the space environments have been reviewed elsewhere (Milojevic & Weckwerth, 2020).

With the advent of the omics era, a number of genomes of extremophiles have been sequenced, and their adaptive physiology have been discerned. Furthermore, metagenomics enables reconstructions of extremophiles genomes, bypassing the laborious steps of isolation and cultivation, and sometimes even guiding tailor‐made isolation procedures for the microorganisms of interest (Katayama et al., 2020). This greatly pave the way to the reconstruction of GEMs of extremophiles as presented in Table 1. These studies demonstrate the great potential of GEMs in exploring metabolic fluctuations under extreme conditions, with implications for astrobiological studies. For instance, a comprehensive GEM of *Parageobacillus thermoglucosidasius* NCIMB 11955 accurately predicted the utilization of 22 carbon sources under aerobic conditions and identifying a bottleneck in anaerobic metabolism. This model offers valuable insights into the minimal required nutrients for anaerobic growth (Mol et al., 2021). Another significant contribution is the first GEM reconstructed for the Antarctic microbial strain *Pseudoalteromonas haloplanktis* TAC125. Through validation against experimental data, this model reveals temperature‐dependent changes in gene expression and the cellular metabolic fluxes of this cold‐adapted bacterium (Fondi et al., 2015). Additionally, a GEM of the hyperthermoacidophilic crenarchaeon *Sulfolobus solfataricus*, accurately predicting growth on different carbon sources and reflecting its metabolic capabilities (Ulas et al., 2012). Furthermore, the GEM for *Shewanella piezotolerans* strain WP3 was reconstructed and exhibits unique metabolic features compared to other *Shewanella* species. This model demonstrates its energy conservation mechanisms and adaptation to fluctuating carbon sources in the deep sea (Dufault‐Thompson et al., 2017).

Isolation and cultivation of extremophiles are critical for in‐depth analysis and validation of metabolic models. However, these processes pose significant challenges due to the unique requirements of extremophilic organisms. For instance, the cultivation of piezophiles necessitates specialized equipment and complex techniques to provide pressures of at least 40 MPa, which are essential for their growth (Dalmasso et al., 2016; Michoud & Jebbar, 2016). Similarly, thermophiles thrive at temperatures above the melting points of conventional microbiological gelling agents, necessitating the use of alternative substances like Gelrite and introducing additional steps in media preparation (Kurr et al., 1991). Moreover, some extremophiles remain uncultivable or yet‐to‐be‐cultivated in standard laboratory settings, limiting our understanding of their diversity, physiological capabilities, and biotechnological potential. Additionally, the functional annotation of prokaryotic genomes, particularly those from non‐model organisms and underrepresented phyla, is incomplete, making it challenging to fully elucidate the functions and roles of genes in their adaptive physiology (Lobb et al., 2020). These limitations highlight that the metabolic modelling of extremophiles for astrobiology study is a complex and challenging task. Overcoming these challenges will require continued research efforts and the development of new methods for collecting and analysing the experimental data.

## CONCLUSION FOR THE APPLICATIONS OF METABOLIC MODELLING IN ASTROBIOLOGY

Metabolic modelling using GEMs has proven to be a powerful tool for studying organisms in silico. Its applications extend to understanding metabolic adaptations of extremophiles in extreme environments and simulating genetic perturbations and environmental changes. The integration of GEMs with other mathematical approaches holds promise for predicting life forms in specific astroenvironments, offering insights into the origins of life and adaptation mechanisms in space. This has implications for redesigning organisms or identifying targets for bioproduction in space using synthetic biology. However, challenges in studying extremophiles and obtaining experimental data for validation, the complexities of organismic biochemical networks, and the limited availability of kinetic parameters must be taken into account for the comprehensive and accurate use of GEMs in this context. The potential of metabolic modelling in astrobiology requires continuous research and innovative approaches to data collection and analysis to fully exploit the opportunities for exploring extremophiles and unravelling metabolic networks.

## AUTHOR CONTRIBUTIONS

**Sahutchai Inwongwan:** Conceptualization (equal); data curation (equal); formal analysis (equal); funding acquisition (equal); supervision (equal); writing – original draft (equal); writing – review and editing (equal). **Nuttapol Noirungsee:** Conceptualization (equal); data curation (equal); formal analysis (equal); validation (equal); writing – original draft (equal); writing – review and editing (equal). **Sakunthip Changkhong:** Conceptualization (equal); formal analysis (equal); writing – original draft (equal). **Kittiya Phinyo:** Conceptualization (equal); visualization (equal); writing – original draft (equal). **Chutipong Suwannajak:** Conceptualization (equal); validation (equal). **Nahathai Tanakul:** Conceptualization (equal); validation (equal).

## CONFLICT OF INTEREST STATEMENT

The authors declare no conflicts of interest.

## Data Availability

All data referenced and utilized in this review article are drawn from publicly available sources as cited within the text and listed in the reference section. No original data or datasets were generated or utilized in this review. The information presented herein is based entirely on previously published studies, articles, books, and other documented sources, and the references for each piece of data are provided.
